# Parents’ Concerns, Behavior, Perception, and Hesitancy Regarding COVID-19 Vaccinations for Children in Central Saudi Arabia

**DOI:** 10.3390/vaccines11101566

**Published:** 2023-10-05

**Authors:** Muhammad Shahid Iqbal, Mohd Naved Khan, Shafqat Qamer, Salah-Ud-Din Khan

**Affiliations:** 1 Department of Clinical Pharmacy, College of Pharmacy, Prince Sattam Bin Abdulaziz University, Al-Kharj 11942, Saudi Arabia; 2 College of Administrative and Financial Sciences, Saudi Electronic University, Riyadh 11673, Saudi Arabia; 3 Department of Basic Medical Sciences, College of Medicine, Prince Sattam Bin Abdulaziz University, Al-Kharj 11942, Saudi Arabia; 4 Department of Biochemistry, College of Medicine, Imam Mohammad Ibn Saud Islamic University (IMSIU), Riyadh 11432, Saudi Arabia

**Keywords:** COVID-19 vaccination, children, parent hesitancy, behavior, perception, Saudi Arabia

## Abstract

In Saudi Arabia, the Ministry of Health (MoH) has implemented strict rules to ensure COVID-19 vaccination uptake by the general public. However, there is hesitancy about COVID-19 vaccination among parents for their children worldwide. We aimed to determine the concerns, behaviors, perceptions, and hesitancy of COVID-19 vaccination among parents for their children in Saudi Arabia. Parents of children aged 5–11 years were included in this cross-sectional study. A total of 1507 responses were obtained using the convenience sampling technique. The data were analyzed using SPSS version 25.0 by applying descriptive and inferential statistics. Of the parents who responded, 74.5% believed that the COVID-19 vaccination could affect the genes of children, and 72.8% believed that the COVID-19 vaccination could have a greater number of positive impacts on the overall health of children. In total, 87% of the parents were satisfied with the vaccination services and effective policies of the MoH, Saudi Arabia. This study concluded that there is a greater need to increase public awareness regarding the beneficial impact of COVID-19 vaccination on the overall health of children. Effective awareness campaigns are also required to provide empirical information to the public that COVID-19 vaccination for children is safe and effective.

## 1. Introduction

Since the declaration of COVID-19 as a global pandemic on 11 March 2020, by the World Health Organization (WHO), COVID-19 vaccination has been demanded worldwide [1]. Indeed, the WHO and the governments of most countries have focused on the quick vaccination of the public to achieve herd immunity against COVID-19. The Ministry of Health (MoH) of Saudi Arabia ensured that all citizens and residents should receive the COVID-19 vaccination, including children [2]. In November 2021, the Saudi government approved the COVID-19 vaccine (Pfizer–BioNTech) to be used for children aged 5–11 years. Different studies have reported that parents have shown hesitance towards the COVID-19 vaccination since its development, which is considered to be a threat to the global COVID-19 pandemic situation [3,4,5,6]. Various studies have also reported that herd immunity is the only effective solution to combat the COVID-19 pandemic. This can be achieved through COVID-19 vaccination at a broader level in the global population [5,6,7]. It has also been reported that immunization achieved through COVID-19 vaccination could play an important role in strengthening the immunity of a person against COVID-19 [8].

Hesitancy towards vaccines refers to delaying or having negative thoughts about receiving a vaccination, resulting in a decreased vaccination rate [8]. In the literature, several studies have reported different reasons why parents have refused, discontinued, or ceased to follow COVID-19 vaccination schedules for their children. These include observations of minor side effects like rashes, disease severity, body aches, etc. [9,10,11,12,13]. In the United States, a COVID-19 vaccination refusal rate of approximately 45% was observed among parents. This was due to a misbelief that the COVID-19 vaccination caused neurodevelopmental disorders in children [14,15,16,17]. Some studies reported that parents had refused to vaccinate their children against COVID-19 because they were healthy and had no symptoms of COVID-19 infection [18,19].

There are numerous factors that are directly associated with COVID-19 vaccine hesitancy among parents, including self-assurance of its safety, self-confidence of its effectiveness, its suitability, and the intensity of its potential side effects in children [6,7,8,9]. In a study conducted by Al Yamani et al. (2022), it was observed that there was a variety of associated multi-faceted and complex factors that were responsible for hesitancy about routine vaccines among parents [20]. These factors included the risk of acquiring an infection after a vaccination shot as well as social, cultural, and religious norms associated with a routine vaccination. The relationship between parents and healthcare providers also influenced the hesitancy about vaccination [20]. In another study by Al Naam et al. (2022), it was observed that only 40.7% of parents were willing to have their children vaccinated against COVID-19, while 59.3% were hesitant to do it [21]. Ghosh et al. (2022) observed that 58% of parents were concerned that routine vaccines could impact the hormonal balance of their child which can affect their puberty [22]. A gap in the literature about the potential benefits of vaccination is also another factor that affects parents’ decisions about vaccinations [21,22,23].

There are other major determinants or factors directly influencing parental decisions regarding COVID-19 vaccinations for their children. First and foremost is social media, which plays a significant role in the development of hesitancy for the COVID-19 vaccine among parents [23,24]. A study undertaken by Almuqbil et al. (2023) reported that social media contributed nearly 25% to parental hesitancy regarding the COVID-19 vaccination of their children [23,24,25]. Other factors such as poor perceived immunity, news articles, media reports, personal likes and dislikes, and personal or religious beliefs regarding COVID-19 vaccinations were also significantly responsible for the abhorrence among parents regarding COVID-19 vaccinations for their children [21,22,23]. Due to misinformation regarding COVID-19 vaccine safety and effectiveness, parents have greater hesitancy about administering the COVID-19 vaccine to their children [21,22,23]. There have been rumors in society regarding the potentially harmful effects of the COVID-19 vaccination, like that it might impact puberty, cause sterility, or contain chemicals derived from pigs and monkeys [21,22,23].

On 3 November 2021, the Saudi Food and Drug Authority (SFDA) officially approved the Pfizer–BioNTech COVID-19 vaccine for children aged between 5 and 11 years [24]. According to the WHO and United Nations International Children’s Emergency Fund (UNICEF) reports, parental hesitancy regarding COVID-19 vaccination for their children is present in more than 90% of countries across the globe [25]. In some studies, COVID-19 vaccine hesitancy among parents ranged from 35% to 50% [26,27,28]. Several studies reported the concerns and hesitancy of parents towards COVID-19 vaccination for their children in different parts of the world, such as Italy, Australia, France, Hong Kong, UK, UAE, and Canada [9,10,11,12,13,14,15,16,17,18,19]. However, in the literature, only a few studies are evident from Saudi Arabia regarding parents’ hesitancy to give COVID-19 vaccinations to their children. Therefore, this study aimed to determine parents’ concerns, behaviors, perceptions, and hesitancy regarding COVID-19 vaccination for children in central Saudi Arabia.

## 2. Materials and Methods

### 2.1. Study Design and Study Setting

A cross-sectional approach was used for the study design. Data were collected from February to August 2022. All aspects of the study protocol, including information on the background of the parents, were strictly confidential and used for research purposes only.

### 2.2. Sampling Strategy and Study Site

A convenient sampling technique was used to obtain the required sample size from central Saudi Arabia.

### 2.3. Sample Size

Riyadh province is located in the central part of Saudi Arabia and has an approximate population of 8 million. The study sample size was calculated after adjusting for a potential non-response rate (40%) using the formula below, as described in various studies [29,30,31]:

$$n=\frac{N}{1+{\mathrm{Nd}}^{2}}\;\mathrm{and},$$

$$n_{n}=\frac{n}{1-d}$$

where n is the required sample size, N is the total population size, d is the error margin (0.05 is usually an ideal value when the confidence interval/level is 95%), n_n_ is the sample size (after consigning a non-response rate), n is the earlier calculated sample size, and d is the non-response rate. According to the above calculation, the final estimated sample size for this study (with a 40% non-response rate) was approximately 598; we obtained 1578 samples. After the data collection, 71 samples were excluded because of incomplete or missing responses. Statistical calculations were applied to 1507 samples.

### 2.4. Inclusion and Exclusion Criteria

The study inclusion criteria were adult parents aged ≥18 years who had at least 1 child aged from 5 to 11 years old. All residents in the central region of Saudi Arabia who were 18 years or older and agreed to participate in the study were eligible to be included. Prior to the start of the study, participants were asked about their willingness (consent) to be involved in the study. Those who did not complete the research tool or who did not agree to participate were excluded from the study.

### 2.5. Study Tool

A research tool was designed after an extensive literature review [32,33,34,35]. This was customized and modified according to the aim and objectives of the study. To ascertain the content and face validity, the study tool was validated and pre-tested by five healthcare providers and two parents with children who had previously been administered COVID-19 vaccinations. The included healthcare providers had experience in the general vaccination of children as well as COVID-19 vaccinations and in treating and managing childhood diseases (two pediatric physicians, two immunologists, and one clinical pharmacist working in a COVID-19 vaccination facility).

Close-ended questions were added to the study tool. A pre-test (pilot study) was also conducted among 30 randomly selected parents to ensure the reliability, validity, and understandability of the questions and to eliminate bias in the data. The results of the pre-test were not included in the final data. The research tool was divided into four different sections: the demographics (Section 1) and three other sub-sections (sub-scales). Section 1 comprised sociodemographic attributes of the parents, including age, marital status, gender, etc. Section 2 comprised concerns of parents regarding vaccinations (10 items). The third part comprised behaviors and perceptions of parents towards the COVID-19 vaccination of children (8 items). The fourth part comprised the hesitancy of parents towards the COVID-19 vaccination of children (8 items). Cronbach’s alpha was determined for each sub-scale and in summation (grouped/combination) of the three subscales to determine the reliability of the research tool, similar to numerous other studies reported in the literature [23,36]. Cronbach’s alpha values of 0.838, 0.911, and 0.898 were obtained for Section 2, Section 3 and Section 4 of the research tool, respectively. An overall Cronbach’s alpha value of 0.882 was also obtained for the entire research tool (26 items). This ascertained the internal consistency (reliability) of the newly developed study tool.

Originally, the study tool was designed in English. Then it was translated into Arabic (a locally spoken language) by two proficient Arabic speakers who were content/subject matter experts. This version was then re-verified using a reverse translation method by two bilingual expert reviewers who were proficient Arabic and English speakers. The study tool was revised; all inconsistencies from the translations and the results of the pilot study were addressed (where needed) to finalize the tool and ensure its suitability to obtain data from the target population. There was no difficulty observed in reading and understanding the final developed study tool, and it was deemed appropriate for use to obtain the required data.

### 2.6. Data Collection

The research tool was distributed to the parents of children (aged 5–11 years) using various methods using different social media platforms and in hard copies (where applicable). Study participants were also approached at numerous diverse locations, such as shopping malls, mega malls, schools, supermarkets, parks, small markets, gardens, toy shops, primary healthcare centers, pharmacies, hospitals, restaurants, and other allied healthcare facilities. A barcode for the study tool was also generated and displayed at the entrances of these locations to obtain a greater number of responses. A research tool link was also shared within known groups on different social media platforms, such as IMO, Telegram, and WhatsApp. Study participants had the option to participate or decline the request. Participants were also informed that all collected data were strictly confidential.

### 2.7. Ethical Concerns

A brief explanation was provided on the first page of the study tool regarding the aim and objectives of the study, along with the informed consent declaration. It was also stated that participation was voluntary. To enhance data confidentiality, personal data such as names and addresses were not collected. The data of all participants were anonymous; respondents were asked to take their time to understand the questions and respond honestly. The participants were asked to complete the questionnaire once only, i.e., either online or using a paper-based format.

### 2.8. Statistical Analyses

The collected data were entered and processed using MS Excel and SPSS version 25.0. The data were analyzed using descriptive and inferential statistics. The descriptive statistics (frequency and percentage) were applied to the data obtained. For further analysis, Pearson’s chi-squared test was applied to determine the opinion of the parents regarding the positive impact of the COVID-19 vaccination on children’s overall health. A *p*-value less than 0.5 was considered to be statistically significant.

## 3. Results

Table 1 demonstrates the sociodemographic attributes of parents and their opinions regarding the positive impact of the COVID-19 vaccination on children’s overall health. From the obtained results, 537 (35.6%) had the opinion that the COVID-19 vaccination could have a more positive impact on children’s overall health, whereas 435 (28.9%) stated that the COVID-19 vaccination would not have any positive impact, and 535 (35.5%) did not know or were not sure. Among the study participants, the number of male parents demonstrating their opinion was higher (1101 (70.9%) vs. 406 females (29.1%)). In the age group 31–40 years, 241 respondents (33.1%) believed that the COVID-19 vaccination would have a positive impact on children’s health, while only 51 (14.8%) believed that the COVID-19 vaccination would not have any impact on children’s overall health. Married parents showed greater concern compared with single/separated parents (1278 vs. 229 participants, respectively). Among the married parents, 450 (83.8%) believed that the COVID-19 vaccination would have a positive impact on the overall health of children.

Most of the parents with pre-university qualifications (324 (57.5%)) perceived that the COVID-19 vaccination would have a positive impact on the health of children compared with those who had university-level education (74 (13.2%)). The majority of parents who had healthcare personnel as a family member (92 (16.3%)) believed that the COVID-19 vaccine would have a positive impact on children’s health compared to those who did not have a healthcare member in the family (474 (83.7%)). Among the total employed parents (n = 1007), 362 (63.5%) agreed with the positive impact of the COVID-19 vaccination compared with the unemployed or retired participants (n = 500). Among them, 208 (36.5%) agreed with the positive impacts of the COVID-19 vaccination. Among the parents who were healthcare providers themselves (n = 331), 228 believed that the COVID-19 vaccination would have a positive impact, compared to 47 (13.2%) who believed that there would be no positive impact on children. Statistically significant differences were observed among several socio-demographic characteristics and the opinion of the included parents regarding the positive impact of COVID-19 vaccination on children’s overall health.

Table 2 shows the questions asked to understand the concerns of parents about COVID-19 vaccination for children. Out of the total participants, 1123 (74.5%) believed that vaccines could affect the genes of children; 212 (14%) did not know about any effects; and 172 (11.5%) believed that they would not affect the genes of children. When participants were asked about the challenges faced by them regarding the COVID-19 vaccination, the majority (993 (65.9%)) stated that they did not face any challenges obtaining the vaccination. Only 496 (32.9%) experienced some difficulties in obtaining a COVID-19 vaccination, especially booking/reserving online slots. Of the participants, 621 (41.3%) shared a concern that they had observed some minor side effects on children after a COVID-19 vaccination, 391 (25.9%) shared that they did not observe any side effects, and 495 (32.8%) did not remember or were not sure. It was observed that 417 (27.7%) participants thought death or casualty could occur after a COVID-19 vaccination, and 705 (46.8%) believed that such consequences would not occur at all. The majority of participants (1329 (88.1%)) were satisfied with the Saudi Arabian MoH vaccination system; only 145 (9.7%) were not satisfied (due to a long waiting time for the booking of online slots) and 33 (2.2%) did not know. A total of 724 (48%) participants reported that they were advised by their friends/family members to have the COVID-19 vaccination for their children, and 431 (28.6%) stated that they were not advised by their friends/family members to take the COVID-19 vaccination for their children, whereas 352 (23.4%) reported that they were not sure whether their family members recommended them or not for the COVID-19 vaccination for their children. Approximately 10.7% of the participants believed that COVID-19 vaccinations could affect the respiratory system of children and 42.9% responded that they had themselves seen major side effects of the COVID-19 vaccines in adults. Additionally, only 24% thought that the COVID-19 vaccination was not safe, whereas 48.5% thought it was safe, and 27.5% were not aware of its safety.

Table 3 shows the behavior and perception of parents regarding COVID-19 vaccinations for children. Out of 1507 participants, 692 (45.9%) stated that they had observed some minor side effects of the COVID-19 vaccine in children, whereas 416 (27.6%) shared that they did not see any side effects in children. The majority of participants (969 (64.3%)) stated that their children had received flu vaccine shots; 275 (18.2%) had not, and 263 (17.5%) were not sure. Approximately 52% of parents believed that children would not become COVID-19 positive after vaccination; 22.1% were afraid that children would be COVID-19 positive even after the vaccination. It was observed that 578 (38.3%) parents believed that those children should not receive a COVID-19 vaccination if they had already had a COVID-19 infection; 412 (27.3%) believed that such children should also receive a COVID-19 vaccination even after a COVID-19 infection. In total, 73.3% of the parents believed that there is a scarcity of scientific data regarding COVID-19 vaccination safety, especially among children, and, therefore, it should not be administered to children. We noted that 72.8% of the parents perceived that the COVID-19 vaccination will have beneficial effects on children’s health, and 65.8% of the parents believed that the COVID-19 vaccination is effective in controlling and protecting against a COVID-19 infection; only 12.4% believed that it will not be effective. Approximately 80.7% of participants believed that a COVID-19 vaccination would cause some hormonal imbalances in children but that, in the future, they could enjoy a good quality of life; 9.6% were not sure about it.

Table 4 demonstrates the hesitancy of parents regarding COVID-19 vaccination for their children. We observed that the majority of the parents (989 (65.6%)) were not hesitant about vaccinating their children, and 1215 (80.6%) parents believed that a COVID-19 vaccination was crucial for the future health of their children. In total, 1352 (89.7%) participants shared that they were willing to have a COVID-19 vaccination for their children; 447 (29.7%) believed that a COVID-19 vaccination would not affect the quality of life of their children, whereas 237 (15.7%) believed that it would affect the quality of life, and 823 (54.6%) did not know about it. Some parents (21.3%) responded that they were hesitant regarding the COVID-19 vaccination because they had heard about some side effects. In total, 87% believed that MoH services for the COVID-19 vaccination program were extremely satisfactory.

## 4. Discussion

The COVID-19 vaccination is considered to be the only safe and effective method to combat the COVID-19 pandemic. Therefore, efforts have been made on a larger scale in all countries to achieve the goal of herd immunity [24,37]. After the declaration from the WHO regarding the suitability of the COVID-19 vaccination for children, parents have been advised to take the COVID-19 vaccination for their children. Like other countries, the MoH, Saudi Arabia, has implemented strict, but public-friendly, rules regarding the COVID-19 vaccination for children [38]. With this study, we aimed to determine the concerns, behaviors, perceptions, and hesitancy of parents in Saudi Arabia regarding the COVID-19 vaccination for children. It has been observed that a decline in childhood COVID-19 vaccinations could occur in the upcoming years because of several misconceptions about the COVID-19 vaccines’ side effects and adverse effects. Due to several circulating myths or certain strict beliefs of the public across the globe (religious, social, or cultural), the acceptance of COVID-19 vaccination for children by parents is slow or declining [39]. Therefore, there is a greater need to explore the actual facts and figures about the hesitancy of COVID-19 vaccination among parents for their children and to analyze the possible reasons behind it.

There was a statistically significant difference observed between various sociodemographic attributes and the opinion of parents regarding the positive impact of COVID-19 vaccination on children’s overall health. We observed that male parents (n = 1101) were more open in expressing their opinions and participating compared with female parents (n = 406). It was further noted that parents from the age group 31–40 years were more positive towards COVID-19 vaccination compared with other age groups (specifically, older parents). Our study results were in agreement with two other studies, namely Aedh et al. (2022) and Khatatbeh et al. (2022), which reported similar results. In both of these studies, parents younger than 40 years were less hesitant about COVID-19 vaccination of their children [40,41]. In our study results, parents who had healthcare professionals in their families showed a more positive attitude towards COVID-19 vaccination compared with those who did not have healthcare professionals in their families. It was also noted that parents with 1–2 children had more positive opinions about COVID-19 vaccination compared with those who had >2 children. These findings corroborated the findings of another study [42].

The results of the current study showed that the majority of parents in Saudi Arabia were not that hesitant about the COVID-19 vaccination for their children and believed them to be safe and effective. Several parents were a little apprehensive about the COVID-19 vaccination side effects and believed that the COVID-19 vaccination might have some negative impacts on the overall health of the children. Another interesting finding was that, despite COVID-19 vaccine hesitancy, the majority of included parents had already vaccinated or were willing to vaccinate their children. This may be explained by the rigorousness of the policies of the MoH, Saudi Arabia, regarding COVID-19 vaccination for children. Indeed, these measures were beneficial to better save and safeguard the population in Saudi Arabia. It was also observed that the majority of the parents (989 (65.6%)) were not hesitant regarding the COVID-19 vaccination of their children, and 1215 (80.6%) believed that the COVID-19 vaccination was crucial for the health of their children. In total, 1352 (89.7%) parents shared that they were willing for their children to have the COVID-19 vaccination. Of these, 447 (29.7%) believed that the COVID-19 vaccination would not affect the quality of life of their children, 237 (15.7%) believed that it would, and 823 (54.9%) were not sure about it. Some parents (with a frequency of 21.3%) responded that they were a bit hesitant regarding the COVID-19 vaccination because they had heard about its side effects. In total, 87% believed that MoH services were satisfactory; only 11.1% considered them to be unsatisfactory.

We observed that parents were a little hesitant about vaccinating their children because they believed that it would affect the genes as well as the puberty of the children. Some parents believed that the COVID-19 vaccination could be more harmful to children than adults because it could cause hormonal imbalances in their bodies, especially those that control puberty. Jacobs et al. (2022) also observed this reason to be behind the disagreement between the decisions of parents regarding the COVID-19 vaccination of their children and concerned healthcare authorities [42]. A few of the parents were of the opinion that the immune system of a child is not as developed as that of an adult and that they would have greater hormonal changes in their body due to the COVID-19 vaccination [42,43]. This perspective of parents is likely due to plenteous fake news spreading on social media regarding COVID-19 vaccines, despite the approval and clearance provided by the WHO [43,44,45]. This is similar to the findings of Talabi et al. (2022) that those who were exposed to fake news regarding COVID-19 vaccines demonstrated more negative behavior and had false perceptions of COVID-19 vaccination [43]. Another reason was that a large proportion of parents who had previously gotten their children vaccinated had observed certain side effects in their children, spreading unwanted fear among other parents [44,45]. Interestingly, most of the parents in the current study believed that the COVID-19 vaccination did not cause any casualties or deaths, which was not the situation when the COVID-19 vaccines had just been introduced to the market. Two other studies also reported similar results that approximately 60% of the parents agreed that the COVID-19 vaccination would protect their children from future COVID-19 infections and complications [46,47]. We also observed that approximately 10.7% of the parents reported that they had a fear of respiratory infections, even after the COVID-19 vaccination. The reason for this could be the rapid genome-changing ability of the SARS-CoV-2 virus.

According to the obtained results regarding the behavior and perception of parents towards COVID-19 vaccination for children, the responses were divided into three categories: “Yes”, “No”, and “Don’t Know/Not Sure”. From the obtained results, all the questions had a *p*-value of <0.001, i.e., smaller than 0.05, demonstrating that the results were statistically significant and not due to chance. Although it is well known that COVID-19 vaccines are the only real (safe and effective) solution to fight against the COVID-19 pandemic, a few parents in the current study believed that the COVID-19 vaccination was not safe and did not provide protection against COVID-19. This may be because it has been claimed that one can get COVID-19 infection even after COVID-19 vaccination. Our results demonstrated that more than half of the parents had permitted flu vaccine shots for their children as directed by the MoH. The reason behind this was the strict rules enforced by the MoH for routine vaccinations in Saudi Arabia.

In the literature, it is reported that higher vaccine hesitancy, lower vaccine uptake, and negative attitudes towards routine vaccines are directly linked to lower socioeconomic status/characteristics of the individuals [48]. These socioeconomic determinants include lower education levels, less social support, low health literacy, lower income, and lower knowledge about vaccine safety and effectiveness [48]. A study reported that lower educational levels were directly linked with negative attitudes towards routine vaccines among parents for their children [49]. Similarly, another study indicated that vaccine hesitancy was low among individuals with higher income and higher education levels [50]. The UNICEF declared that there could be severe consequences to children’s overall health if they are not vaccinated against COVID-19. Obviously, COVID-19 is more severe and life-threatening than many other routine illnesses or abnormalities [51]. Unvaccinated children may be more vulnerable to various severe illnesses than vaccinated children [51,52,53]. Family members of unvaccinated children could easily acquire serious illnesses from the unvaccinated children [51,52]. Such unvaccinated children could contribute to the outbreak of serious life-threatening diseases in their local community, which could easily be widespread. Health-related quality of life could also be compromised in unvaccinated children. The probability of declining life expectancy and school enrollments could also be higher among unvaccinated children [50,51,52,53,54].

Some parents shared that they would have preferred for their children not to be vaccinated against COVID-19, but due to strict policies and worthy potential benefits, they had permitted COVID-19 vaccination for their children. In total, 42.9% of participants shared that they had themselves observed some negative effects of the COVID-19 vaccination in adults (albeit minor); this was one of the reasons why they did not support the COVID-19 vaccination for their children at the current time and preferred to wait for further safety results. These findings were similar to those of two other studies regarding COVID-19 vaccination safety among children [25,32]. The majority of the parents (80.6%) believed that the COVID-19 vaccination was crucial for the health of their children because it would protect them from future COVID-19 infections. Although numerous awareness studies have been published in different countries, the level of COVID-19 health literacy regarding its dangers and life-threatening effects is low worldwide [55]. Healthcare authorities and organizations should ensure that the public has a greater familiarity with health literacy and its advantages when formulating preventive policies in such dangerous pandemics [55].

Healthcare workers (HCWs) also play a vital role in enhancing public awareness of COVID-19 vaccination as they are a trusted source of medical information [56,57]. HCWs can counsel their patients about the long-term benefits of COVID-19 vaccines, which will improve acceptance of COVID-19 vaccination among them as well as the general public [57,58]. This can be done by designing and arranging small public awareness activities, which will further enhance awareness and improve the overall knowledge of society about the safety of the COVID-19 vaccines [56,57,58]. However, on the other side, HCWs are also under pressure to be first-line respondents to the COVID-19 pandemic as they are directly exposed to it [56,57]. This affects their motivation, beliefs, perceptions, and attitudes toward COVID-19 vaccination. Different studies have reported that acceptance of COVID-19 vaccines among HCWs is significantly varied across the globe [59,60]. This variation in acceptance of COVID-19 vaccines ranges from 28% to 95% [59,60,61]. The COVID-19 vaccine sentiments among HCWs were mainly influenced by the different factors/determinants. The type, brand, and manufacturing country were the main factors that affected the choice of COVID-19 vaccines acceptance among HCWs [62,63,64]. Acceptance of COVID-19 vaccines among HCWs is crucial because if they are stressed or not compliant, the COVID-19 vaccination campaigns will fail, which could cause devastating damage to the overall health of society.

### 4.1. Strength and Limitations of the Study

#### 4.1.1. Strength of the Study

The findings of this study provide comprehensive data that may be used to develop, design, and implement various healthcare strategies and policy programs regarding the safety and efficacy of the COVID-19 vaccination and encourage and counsel parents about the healthcare benefits of the COVID-19 vaccination in children.

#### 4.1.2. Limitations of the Study

There were a few limitations to this study. First, there was a risk of recall bias because the responses of participants cannot be independently verified in many cross-sectional studies. Second, as the study was performed in a specific part of Saudi Arabia, it may not be representative of all governorates of the country. Third, no causal implications could be inferred because this was a cross-sectional study. Fourth, this study was conducted using a convenient sampling technique that may have limited the external validity of the obtained results because replicating results is a major concern when using the convenient sampling method.

## 5. Conclusions

Although there was hesitancy among parents regarding the COVID-19 vaccination of the children, the majority accepted the importance of COVID-19 vaccination for the children. The majority of the studied parents were not worried about the COVID-19 vaccination for their children and believed that the COVID-19 vaccination would ultimately help the children enjoy a good quality of life. Although fake news and incorrect information are frequently available on social media, approximately 90% of the parents were very satisfied with the health-beneficial measures taken by the MoH, Saudi Arabia, regarding the COVID-19 vaccination for children. Further empirical information is required regarding the impact of COVID-19 vaccination on the overall health state of children and to increase awareness and reduce hesitancy among parents.

## Figures and Tables

**Table 1:** Sociodemographic attributes and parents’ opinions about the positive impact of COVID-19 vaccination on children’s overall health.

Parents Sociodemographic Variables	Categories	Opinion of Parents Regarding Positive Impact of the COVID-19 Vaccination on Children’s Overall Health						*p*-Value
		Yes		No		Don’t Know/ Not Sure		
		N	%	N	%	N	%	
Gender	Male (n = 1101)	381	70.9	371	85.3	349	65.3	<0.001
	Female (n = 406)	156	29.1	64	14.7	186	34.7	
Age	≤30 (n = 418)	108	14.9	67	19.4	243	55.9	<0.001
	31–40 (n = 384)	241	33.1	51	14.8	92	21.1	
	41–50 (n = 329)	147	20.2	129	37.4	53	12.2	
	>50 (n = 376)	231	31.8	98	28.4	47	10.8	
Marital status	Married (n = 1278)	450	83.8	449	83.9	379	87.1	0.008
	Single/Separated (n = 229)	87	16.2	86	16.1	56	12.9	
Education	No formal education (n = 109)	15	2.7	73	14	21	4.9	<0.001
	Primary (n = 352)	150	26.6	109	20.9	93	21.9	
	Pre-university (n = 862)	324	57.5	291	55.9	247	58.5	
	University (n = 184)	74	13.2	48	9.2	62	14.7	
Have a healthcare provider in the family	Yes (n = 240)	92	16.3	63	15.3	85	16.1	<0.001
	No (n = 1267)	474	83.7	350	84.7	443	83.9	
Employment	Employed (n = 1007)	362	63.5	318	73.1	327	65.1	0.004
	Retired/Unemployed (n = 500)	208	36.5	117	26.9	175	34.9	
Monthly income	≤5000 SAR (n = 606)	279	41.3	154	38.8	173	39.8	<0.001
	5001–10,000 SAR (n = 816)	362	53.6	222	55.9	232	53.3	
	>10,000 SAR (n = 85)	34	5.1	21	5.3	30	6.9	
Are you a healthcare provider?	Yes (n = 331)	228	31.8	47	13.2	56	12.9	<0.001
	No (n = 1176)	488	68.2	309	86.8	379	87.1	
Number of children	1–2 (n = 999)	454	56.3	184	69.4	361	82.9	0.007
	>2 (n = 508)	353	43.7	81	30.6	74	17.1	

**Table 2:** Parents’ concerns regarding COVID-19 vaccination in children.

Questions	Yes		No		Don’t Know/ Not Sure		*p*-Value
	N	%	N	%	N	%	
Do you think that COVID-19 vaccination could affect children’s genes?	1123	74.5	172	11.5	212	14	<0.001
Do you think COVID-19 vaccination has more side effects in children than adults?	913	60.5	219	14.6	375	24.9	<0.001
Did you face any challenges and difficulties in getting COVID-19 vaccination?	496	32.9	993	65.9	18	1.2	<0.001
Did any of your children face any minor side effects of COVID-19 vaccination?	621	41.3	391	25.9	495	32.8	0.015
Do you believe that a causality/death can happen due to COVID-19 vaccination?	417	27.7	705	46.8	385	25.5	<0.001
Are you satisfied with the MoH vaccination system?	1329	88.1	145	9.7	33	2.2	<0.001
Have your family members/friends advised you not to vaccinate your children from COVID-19 vaccination?	724	48	431	28.6	352	23.4	<0.001
Do you have any fear of respiratory infections after getting COVID-19 vaccination?	160	10.7	394	26.1	953	63.2	<0.001
Did you see any major side effects of COVID-19 vaccination in your own eyes in adults?	647	42.9	382	25.4	478	31.7	<0.001
I strongly believe that COVID-19 vaccination is not safe and does not provide good protection to children.	362	24	731	48.5	414	27.5	<0.001

**Table 3:** Parents’ behavior and perception regarding COVID-19 vaccination in children.

Questions	Yes		No		Don’t Know/ Not Sure		*p*-Value
	N	%	N	%	N	%	
I have seen some minor side effects of COVID-19 vaccination in children.	692	45.9	416	27.6	399	26.5	<0.001
I gave my children seasonal flu vaccine shots last year, as advised by MoH.	969	64.3	275	18.2	263	17.5	<0.001
I am afraid that after COVID-19 vaccination, the children will become COVID-19 test positive.	332	22.1	784	52	391	25.9	<0.001
I prefer not to give COVID-19 vaccination to children if they have already had COVID-19.	578	38.3	412	27.3	517	34.4	0.009
I prefer not to give the COVID-19 vaccination to children as there is a scarcity of scientific data regarding its safety.	1104	73.3	278	18.4	125	8.3	<0.001
I am confident that COVID-19 vaccination will have a beneficial impact on the children’s health.	1098	72.8	353	23.5	56	3.7	<0.001
I believe that the available COVID-19 vaccination is not very effective in controlling COVID-19 infections in children.	185	12.4	993	65.8	329	21.8	<0.001
I feel that after the COVID-19 vaccination, there might be some hormonal imbalance in children, but overall, they will enjoy a good quality of life.	1216	80.7	146	9.7	145	9.6	<0.001

**Table 4:** Parents’ hesitancy regarding COVID-19 vaccination in children.

Questions	Yes		No		Don’t Know/Not Sure		*p*-Value
	N	%	N	%	N	%	
Are you hesitant to have your children COVID-19 vaccinated?	414	27.5	989	65.6	104	6.9	<0.001
I think COVID-19 vaccination is too crucial for my children’s future health.	1215	80.6	148	9.8	144	9.6	<0.001
I am willing to have my children COVID-19 vaccinated.	1352	89.7	143	9.5	12	0.8	<0.001
I believe that COVID-19 vaccination will not affect overall quality of life of my children.	447	29.7	237	15.7	823	54.6	<0.001
I am hesitant about COVID-19 vaccination for my children because I heard it has some side effects.	321	21.3	742	49.2	444	29.5	<0.001
I believe that COVID-19 vaccination would not affect the puberty of my children.	698	46.4	397	26.3	412	27.3	<0.001
Do you recommend others for COVID-19 vaccination to their children?	520	34.5	395	26.2	592	39.3	0.005
Are you satisfied with the COVID-19 vaccination services provided by MoH?	1310	87	169	11.1	28	1.9	<0.001

## Data Availability

Data is available upon a reasonable request.
